# Huaier Induces Immunogenic Cell Death *Via* CircCLASP1/PKR/eIF2α Signaling Pathway in Triple Negative Breast Cancer

**DOI:** 10.3389/fcell.2022.913824

**Published:** 2022-06-16

**Authors:** Chen Li, Xiaolong Wang, Tong Chen, Wenhao Li, Xianyong Zhou, Lishui Wang, Qifeng Yang

**Affiliations:** ^1^ Department of Breast Surgery, General Surgery, Qilu Hospital, Cheeloo College of Medicine, Shandong University, Jinan, China; ^2^ Department of Clinical Laboratory, Qilu Hospital, Cheeloo College of Medicine, Shandong University, Jinan, China; ^3^ Department of Pathology Tissue Bank, Qilu Hospital, Cheeloo College of Medicine, Shandong University, Jinan, China; ^4^ Research Institute of Breast Cancer, Shandong University, Jinan, China

**Keywords:** Huaier, ICD, circRNA, PKR, er stress

## Abstract

Triple-negative breast cancer (TNBC) is the most lethal breast cancer subtype owing to the lack of targeted therapeutic strategies. Immunogenic cell death (ICD), a modality of regulated cancer cell death, offered a novel option for TNBC *via* augmenting tumor immunogenic microenvironment. However, few ICD-inducing agents are currently available. Here, we showed that *Trametes robiniophila Murr* (Huaier) triggered ICD in TNBC cells by promoting cell surface calreticulin (CRT) exposure, and increasing release of adenosine triphosphate (ATP) and high-mobility group protein B1 (HMGB1). Co-culturing with Huaier-treated TNBC cells efficiently enhanced the maturation of dendritic cells (DCs), which was further validated via cell-based vaccination assay. In the xenograft mouse model, oral administration of Huaier led to tumor-infiltrating lymphocytes (TILs) accumulation and significantly delayed tumor growth. Besides, depletion of endogenous T cells obviously abrogated the effect. Mechanically, Huaier could elicit endoplasmic reticulum (ER) stress-associated ICD through eIF2α signaling pathway. Further studies revealed that circCLASP1 was involved in the Huaier-induced immunogenicity by binding with PKR in the cytoplasm and thus blocking its degradation. Taken together, we highlighted an essential role of circCLASP1/PKR/eIF2α axis in Huaier-induced ICD. The findings of our study carried significant translational potential that Huaier might serve as a promising option to achieve long-term tumor remission in patients with TNBC.

## Introduction

According to the latest Global Cancer Statistics, with 2.3 million newly diagnosed cases (11.7%), female breast cancer has exceeded lung cancer as the most frequently occurring cancer type across the world (Sung et al., 2021). Accounting for approximately 15%–20% of all breast cancer patients, triple negative breast cancer (TNBC) was typically associated with aggressive behavior, unsatisfied response rates and poor prognosis (Keenan and Tolaney, 2020). Due to the absence of endocrine receptors and HER2, limited treatment options were currently available for TNBC patients (Mu et al., 2021).

Nature is a treasure trove for the investigation of anticancer agents. Traditional Chinese Medicine (TCM), a complicated natural herbal medicine system, was distinguished by the features of multiple targets, low adverse effects and significant curative effect (Wang et al., 2021). Recent researches revealed an emerging role of TCMs in the treatment of various malignancies. Huoxue Yiqi Recipe-2 (HYR-2) was demonstrated to inhibit lung cancer via suppressing the expression of PD-L1 (Teng et al., 2020). Aqueous extract from Astragalus membranaceus, also known as Huangqi, could considerably slow tumor growth and initiate cell apoptosis by PI3K/AKT/mTOR axis (Zhou et al., 2018). As for TNBC treatment, Xihuang pill could manipulate the stemness of tumor cells (Zhang et al., 2021). Meanwhile, ethanol crude extracts from B. javanica seed (BJE) were reported to reduce autophagy through PI3K/Akt/mTOR signaling networks in TNBC patients (Chen et al., 2020).

*Trametes robiniophila Murr* (Huaier) was a sandy beige mushroom which belonged to Class Hymenomycetes, Phylum Basidiomycota (Li C. et al., 2020). Being used as a medicinal ingredient in China for approximately 1,600 years, Huaier had been revealed to play a vital role in several types of cancers (Li C. et al., 2020). A nationwide multicenter clinical trial indicated that combined use of Huaier granule as adjuvant therapy could obviously prolong the recurrence-free survival in hepatocellular carcinoma patients (Chen et al., 2018). Recent years, mounting evidence demonstrated the key role of Huaier in manipulating the tumor microenvironment (TME). We have previously proved that Huaier substantially attenuated breast cancer progression *via* regulating the recruitment and polarization of tumor associated macrophages (TAMs) (Li et al., 2016). Recent study also revealed that Huaier could promote maturation of dendritic cells (DCs), with potent ability to evoke Th1 immune response (Pan et al., 2020). However, the underlying mechanisms of Huaier were still largely unclear.

Immunogenic cell death (ICD) is a form of regulated cell death which is characterized by the emission of tumor-associated antigens (TAAs) and danger-associated molecular patterns (DAMPs) from dying cancer cells (Jeong et al., 2021). As an initial step in the ICD signaling cascades, immunogenicity of tumor cells could be evoked through generation of reactive oxygen species (ROS) which induce endoplasmic reticulum (ER) stress (Deng et al., 2020). Recent years, induction of immunogenic cancer cell death (ICD) is emerged as one of the most encouraging ways to establish a long-term cancer immunity (Alzeibak et al., 2021). Teniposide, a DNA topoisomerase II inhibitor, was reported to effectively suppress tumor progression through induction of ICD via cGAS/STING pathway (Wang et al., 2019). Moreover, SR-4835, a CDK12/13 specific inhibitor, could elicit ICD and initiate a T-cell-dependent tumor elimination in breast cancer (Li Y. et al., 2020). Thus, induction of ICD is a pivotal way to impede cancer progression.

In the study, we investigated the anti-tumor effects of Huaier with regard to the possible involvement of ICD. Huaier-treated breast cancer cells showed substantially enhanced cell surface expression of CRT and increased release of ATP and HMGB1. Further investigations showed that Huaier-treated TNBC cells drastically facilitated the activation of DC cells which could be verified in a vaccine setting. Oral administration of Huaier strikingly augmented the infiltration of lymphocytes and inhibited tumor growth *in vivo*. The interaction between ICD and circRNAs was firstly demonstrated in the study.

## Materials and Methods

### Animals and Ethics Approval

The animal experiments were performed in strict accordance with the Guidelines for the Care and Use of Laboratory Animals of Shandong University. All animal procedures were approved by the Ethical Committee on Scientific Research of Shandong University Qilu Hospital (license number: KYLL-202107-123-2).

### Cell Culture and Drug Reagents

Mouse breast cancer cell line (4T1) and human breast cancer cell lines (MDA-MB-231, MDAMB-468) were obtained from American Type Culture Collection (ATCC, Manassas, VA, United States). All cell lines were routinely maintained in the Dulbecco’s modified Eagle’s medium (DMEM)/high glucose medium (Hyclone, UT, United States) contained with 10% fetal bovine serum (FBS) (Gemini, CA, United States), and 1% penicillin-streptomycin (Macgene, Beijing, China) at a 37°C cell culture incubator with 5% CO_2_.

Bone marrow (BM) cells were obtained using a previously described method (Lutz et al., 1999; Li et al., 2019). BALB/c female mice were euthanized via inhalation of 5% isoflurane before sacrificed through cervical dislocation. Femur and tibia were then separated from the surrounding muscle tissue. After immersion in 75% ethanol for 5 min, intact bones were then washed with PBS. Both ends of bones were cut with scissors and marrow was flushed into petri dishes. Primary cells were cultured in the RPMI-1640 medium (Hyclone, UT, United States) supplemented with 40 ng/ml recombinant mouse GM-CSF (PeproTech, Cranbury, NJ, United States) and 20 ng/ml recombinant mouse IL-4 (PeproTech, Cranbury, NJ, United States) for 5 days to generate CD11c^+^ bone marrow-derived DCs (BMDCs).

Aqueous extraction of Huaier was kindly provided by Gaitianli Medicine Co. Ltd (Jiangsu, China). Preparations of Huaier were described in our previous study (Li C. et al., 2020). To yield the active ingredient, the dried fungus of Huaier was extracted by hot water in triplicate after grinding. Sevag method was utilized to eliminate unconjugated proteins and amino acids. Dialysis was then conducted to get rid of micro molecules in the aqueous solution. After precipitated by ethanol, Huaier ointment was further centrifuged and dehydrated for refinement with 1.5%–1.8% proteoglycan yield. To ensure the consistency of the active ingredient and avoid the variation between batches, the chromatographic fingerprint analysis was employed for quality control.

### Identification of Differentially Expressed Genes

RNA quality and quantity was assessed using a Bioanalyzer 2100 (Agilent Technologies, CA, United States) and RNA sequencing was performed on the Illumina HiSeq platform with a 150-bp paired-end approach. To identify the differentially expressed mRNAs, Fragments per kilobase million (FPKM) was used to normalize the mRNA expression profile and fold change > 2 was set as criteria. To identify the differentially expressed circRNAs, R package “limma” was utilized (using the criteria of fold change >3 and *p* < 0.05). The corresponding heatmaps were drawn by R package “pheatmap”.

### Bioinformatics Analysis

Gene ontology (GO) Analyses were conducted via online software Database for Annotation, Visualization, and Integrated Discovery (DAVID; https://david.ncifcrf.gov/) with *p* < 0.05 were considered significant. The R packages “ggplot2” and “cowplot” were applied to establish visual gene set enrichment maps with annotations. Differentially expressed circRNAs were visualized via Circos plot using a Perl language.

### Determination of ATP Secretion

Extracellular ATP levels were detected through an Enhanced ATP Assay Kit (Beyotime Biotechnology, Shanghai, China) following the manufacturer’s protocol. The supernatants of breast cancer cells were harvested after treated with increasing concentrations of Huaier at indicated time points. Cell debris was removed by centrifugation. And chemiluminescence derived from ATP was detected on a Microplate Reader (Bio-Rad, Hercules, CA, United States).

### Flow Cytometry Analysis

To determine the cell surface CRT exposure, MDA-MB-231, MDA-MB-468 and 4T1 cells were grown to 50% confluence in the 6-well plates. After treated by Huaier, cells were harvested and washed twice with PBS. Then cells were stained with primary antibody CRT and secondary antibody labeled with Alexa Fluor 488, followed by analysis using flow cytometry (AccuriTM C6 plus; BD Biosciences Pharmingen, San Diego, CA, United States). Data analysis was performed using FlowJo.

For analysis of immune cell populations *in vivo*, minced tumors were enzymatically digested by a cocktail of 2 mg/ml Collagenase IV (C8160, Solarbio, Beijing, China) and 0.2 mg/ml DNAse I (D8070, Solarbio, Beijing, China) in TESCA buffer (G0150, Solarbio, Beijing, China). Single cell suspensions were filtered through a 50-μm nylon cell strainer. Mouse Fc-Receptors were blocked with anti-mouse CD16/32 (TruStain FcXTM PLUS, BioLegend, San Diego, CA, United States) for 10 min before antibody staining. Samples were then incubated with combinations of antibodies against cell surface markers for 30 min at 4°C. The cells were analyzed by flow cytometry (AccuriTM C6 plus; BD Biosciences Pharmingen, San Diego, CA, United States) with data analysis performed using FlowJo.

The following antibodies were used: CD11c (117307, BioLegend, San Diego, CA, United States), CD86 (105005, BioLegend, San Diego, CA, United States), CD3 (100203, BioLegend, San Diego, CA, United States), CD8 (100711, BioLegend, San Diego, CA, United States), CD45 (147711, BioLegend, San Diego, CA, United States), CRT (YT0620, ImmunoWay Biotechnology, Beijing, China) and Alexa Fluor 488 (ZF-0511, ZSGB-Bio, Beijing, China).

### Dendritic Cell Activation Assay

After treated by Huaier for 24 h, 4T1 cells were co-cultured with BMDCs at a 1:1 ratio for 4 h. Subsequently, the cells were stained with antibodies 7-AAD (559925, BD Biosciences Pharmingen, San Diego, CA, United States), CD11c (117307, BioLegend, San Diego, CA, United States) and CD86 (105005, BioLegend, San Diego, CA, United States), and analyzed by flow cytometry. Data analysis was performed using FlowJo.

### Quantitative Real-Time PCR

Total RNA was extracted with the RNA-easy Isolation Reagent (R701-01, Vazyme, Nanjing, China). Total RNA was finally suspended in 20 µL of RNase-free water. The purity and quality of the isolated RNA was estimated by NanoDrop with A_260_/A_280_ ratio of 1.9–2.0. Genomic DNA (gDNA) was isolated utilizing the TIANamp Genomic DNA Kit (TIANGEN, Beijing, China). To synthesize complementary DNA (cDNA), the PrimeScript reverse transcriptase reagent kit (TaKaRa, Shiga, Japan) was used. qRT-PCR was conducted with SYBR Premix Ex Taq II (TaKaRa, Shiga, Japan) on the Light Cycler 480 II Real-Time PCR System (Roche, Basel, Switzerland). The sequences of primers were listed in Supplementary Table S1. β-actin was utilized as an internal control. Relative RNA abundances were calculated by the standard 2^−ΔΔCt^ method.

### RNA Pull-Down Analysis

The Pierce™ Magnetic RNA-protein Pull-Down Kit (ThermoFisher, MA, United States) was applied to estimate the interaction between circCLASP1 and indicated proteins following the manufacturer’s protocol. Target transcripts were transcribed using MEGAscript™ T7 Transcription Kit (Invitrogen, Carlsbad, CA, United States). Subsequently, Pierce™ RNA 3′ End Desthiobiotinylation Kit (ThermoFisher, MA, United States) was used to label the RNAs. For formation of the probe-magnetic bead complex, biotinylated RNAs were incubated with washed streptavidin magnetic beads for 30 min at room temperature with agitation. The magnetic beads with immobilized probes were incubated with breast cancer cell lysates, followed by washing and elution of the RNA-binding protein complexes. Proteins were obtained and detected by western blotting assay and Mass Spectrometry (MS) analysis.

### RNA Immunoprecipitation Assay

RNA Immunoprecipitation Kit (P0102, Geneseed Biotech, Guangzhou, China) was used according to the manufacturer’s instructions. Briefly, cells at 80% confluence were harvested in RIP lysis buffer. Antibodies against PKR (A19545, ABclonal, Wuhan, China) were applied in the RIP assay, and immunoglobulin G (Normal Rabbit IgG, #2729, Cell Signaling Technology, Danvers, MA, United States) was used as negative control. Extracted RNAs were reverse transcribed and analyzed by qRT-PCR to detect the enrichment rate of circCLASP1.

### Cell Transfection

CircCLASP1 sequence was cloned into the multiple cloning site of pLCDH vector for gain-of-function assays, while siRNA was utilized to knockdown the expression of circCLASP1. The sequence of siRNA was listed in Supplementary Table S2. Empty pLCDH vector and negative control (NC) sequences were used as control. Transfection was conducted using Lipofectamine 2000 (Invitrogen) following the manufacturer’s protocol.

### MTT (3-(4,5-dimethyl-2-thiazolyl)-2,5–diphenyl-2H-tetrazolium Bromide) Assay

Transfected breast cancer cells were seeded into 96-well plates. At the indicated time points, 20 μL of MTT (Sigma, St. Louis, MO, United States) was added into the medium. After another 6 h incubation at 37°C, the culture medium was replaced with 100 μL of DMSO. Absorbance values at 490 and 570 nm were detected via a microplate reader (Bio-Rad, Hercules, CA, United States).

### Protein Isolation and Western Blot

To isolate the protein from the whole cell lysis, after indicated treatment, cells were washed twice by ice-cold PBS and lysed with RIPA Lysis Buffer (P0013B, Beyotime Biotechnology, Shanghai, China) in the presence of protease inhibitors. Cell lysates were centrifugated at 12,000 rpm for 20 min at 4°C to remove cell debris. For separation and isolation of the protein in membrane and cytosol components of cells, Membrane and Cytosol Protein Extraction Kit (Beyotime Biotechnology, Shanghai, China) was used.

The protein samples were separated by electrophoresis in 10% SDS-PAGE and transferred (100 V, 2 h) onto polyvinylidene fluoride (PVDF) membranes (Millipore, Bedford, MA, United States). 5% non-fat milk was used to block non-specific binding sites. After overnight incubation with specific primary antibodies at 4°C and subsequent 2 h incubation with secondary antibodies at room temperature, the protein bands were visualized via ECL (E412-01, Vazyme, Nanjing, China).

The following antibodies were used: β-actin (YM3028, ImmunoWay Biotechnology, Beijing, China), GRP78 (YM1247, ImmunoWay Biotechnology, Beijing, China), eIF2α (YT1507, ImmunoWay Biotechnology, Beijing, China), eIF2α (phospho Ser51) (YP0093, ImmunoWay Biotechnology, Beijing, China), IRE1 (27528-1-AP, ProteinTech Group, Chicago, IL, United States), IRE1 (phospho S724) (EPR5253, Abcam, Cambridge, MA, United States), ATF6A (YT7559, ImmunoWay Biotechnology, Beijing, China), ATF4 (YT1102, ImmunoWay Biotechnology, Beijing, China), ATF4 (phospho Ser219) (YP0503, ImmunoWay Biotechnology, Beijing, China), CRT (YT0620, ImmunoWay Biotechnology, Beijing, China), HMGB1 (YT2183, ImmunoWay Biotechnology, Beijing, China), HSP90 (YT2257, ImmunoWay Biotechnology, Beijing, China).

### Fluorescence *in situ* Hybridization and Immunofluorescence

For detection of the subcellular localization of circCLASP1 in the breast cancer cells, FISH assay was conducted with Fluorescence *in Situ* Hybridization Kit (Cat.10910, GenePharma, Shanghai, China) following the manufacturer’s protocol. Sequence of the probe was listed in the Supplementary Table S3. The sample was incubated in the probe hybridization solution at 37°C overnight with enough humidity.

For the immunofluorescence assay, breast cancer cells were seeded on cell climbing slices in 24 well plates. After indicated treatment, cells were fixed with 4% freshly prepared formaldehyde for 20 min, permeabilized with 0.5% Triton X-100 for 5 min and blocked in 10% goat serum for 1 h at room temperature, followed by overnight incubation with indicated primary antibodies at 4°C. After 2-h incubation with secondary antibodies, DAPI (4′,6′-diamidino-2-phenylindole) was added for DNA staining. Images were taken with a digital camera (Leica, Wetzlar, Germany).

### Animal Vaccination

For the immunization study, 5 × 10^5^ 4T1 cells, which were freeze-thawed 3 times by liquid nitrogen or incubated with Huaier for 24 h, were resuspended in PBS and subcutaneously (s.c.) injected into the left flank of immunocompetent BALB/c mice (female, 4–6 weeks old). PBS was used as negative control. 7 days after the first inoculation, mice in three groups were re-challenged by subcutaneous injection of 5 × 10^5^ live 4T1 cells into the right flank. And the tumor growth was monitored. All mice were euthanized *via* inhalation of 5% isoflurane at a constant flow rate of 1 L/min O_2_ for 3 min before sacrificed through cervical dislocation.

### *In vivo* Tumor Treatment

To study the effects of CD8^+^ T cells in the Huaier treatment, immunocompetent Balb/c mice (female, 4–6 weeks old) were inoculated s.c. at right flanks with 1×10^6^ 4T1 cells in PBS (200 μL). Mice were then randomly divided into four treatment groups and treated with water, Huaier, anti-CD8 antibody and the combination of Huaier and anti-CD8 antibody. Depletion of CD8^+^ T cells was performed using InVivoMAb anti-mouse CD8α (BE0004-1, Bio X Cell, West Lebanon, NH, United States) at days −2, 2, 8 and 14 (100 μg). The Huaier group was given a 100 μL solution containing 50 mg Huaier by gavage every two days, while the control group was given 100 μL water. Tumor dimensions were measured by a caliper every two days and tumor volume was calculated using the following equation: Volume (mm^3^) = width^2^ × length ÷ 2. On day 16, all mice were euthanized and sacrificed by cervical dislocation.

### Statistical Analysis

All assays were performed in triplicate. Statistical analyses in the study were performed with SPSS (version 25.0) and GraphPad Prism 8.0. Significant differences were evaluated by student’s t-test and one-way analysis of variance (ANOVA). *p* < 0.05 was considered statistically significant.

## Results

### Huaier Treatment Induces ICD

**FIGURE 1:**
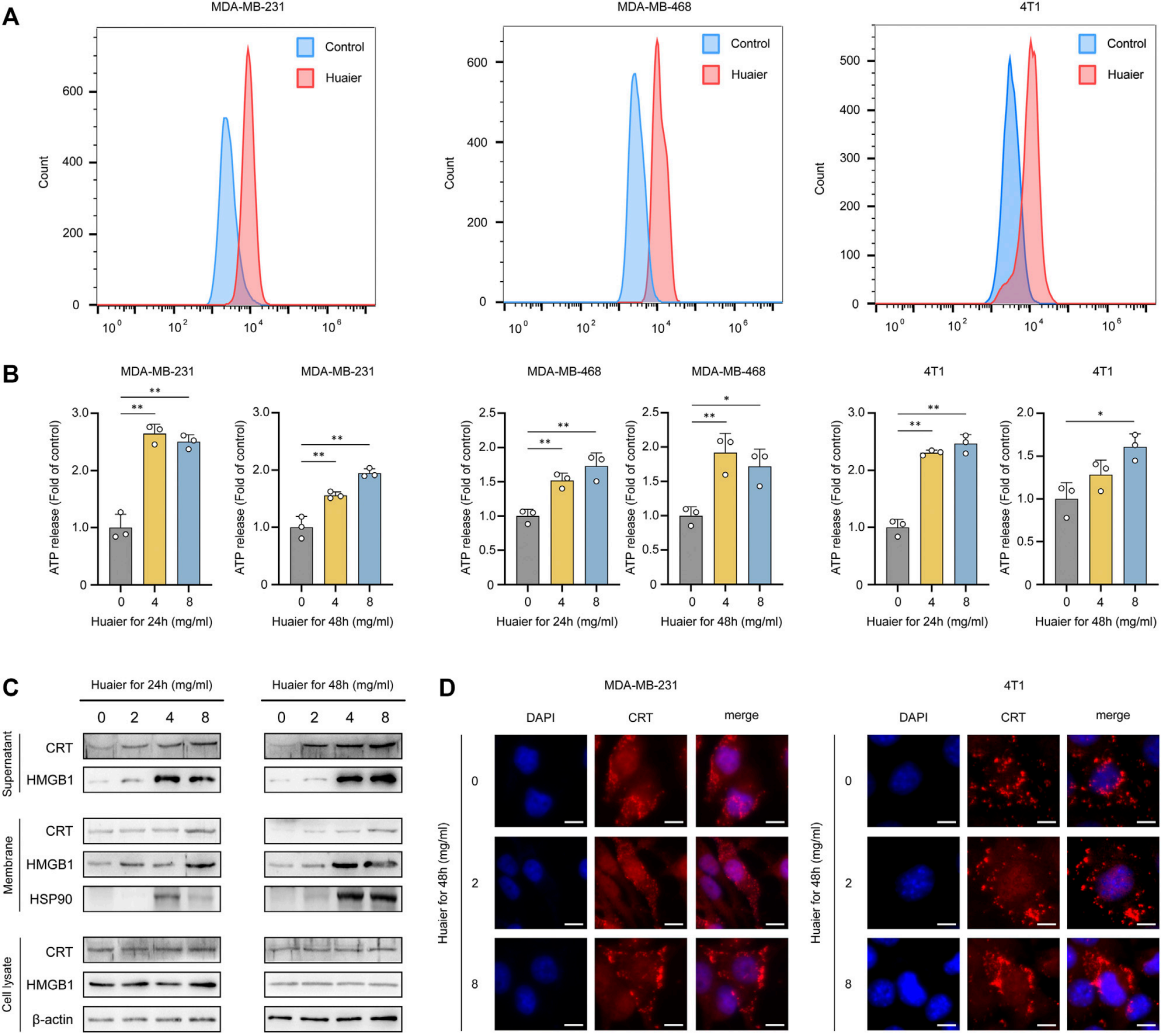
Huaier induces ICD of tumor cells. **(A)** Flow cytometry analysis of surface expression of CRT in breast cancer cells treated with Huaier. **(B)** ATP secretion was detected in the media of TNBC cells treated with Huaier at indicated time and concentration. **(C)** Concentrated supernatants, membranes and cell lysates were collected for western blot. **(D)** CRT translocation was assessed by immunofluorescence staining in MDA-MB-231 and 4T1 cells. Scale bars: 10 μm *p < 0.05; **p < 0.01.

The immunogenic characteristics of ICD are mainly induced by DAMPs, which involve surface-exposed CRT, secreted ATP and HMGB1 (Krysko et al., 2012). TNBC cell lines MDA-MB-231, MDA-MB-468 and 4T1 were treated by Huaier for 24 h, and after incubation, surface expression of CRT was detected by flow cytometry. As shown in Figure 1A, we observed a dramatically increase in CRT levels on the tumor cell surfaces, indicating that the immunogenicity of these cells was substantially facilitated due to the Huaier treatment. Next, to test whether Huaier had impact on the ATP release in tumor cells, we evaluated the culture medium which was collected 24 and 48 h after Huaier-treatment. As shown in the Figure 1B, Huaier could radically up-regulate the extracellular level of ATP in a dose-dependent manner. In addition, the protein levels of ICD markers were also examined. Increased levels of HMGB1 and CRT were observed in the concentrated supernatants in a dose- and time-dependent manner. The protein levels of HMGB1, CRT and HSP90 in cell membrane accumulated after the Huaier treatment (Figure 1C). Consisting with the results, immunofluorescence assays also revealed the translocation of CRT. Remarkably higher levels of CRT were observed on the cell membrane in tumor cells treated by Huaier, while CRT was distributed in the cytoplasm in the control group (Figure 1D).

### Huaier Facilitates Function of DCs in 4T1 Cells

**FIGURE 2:**
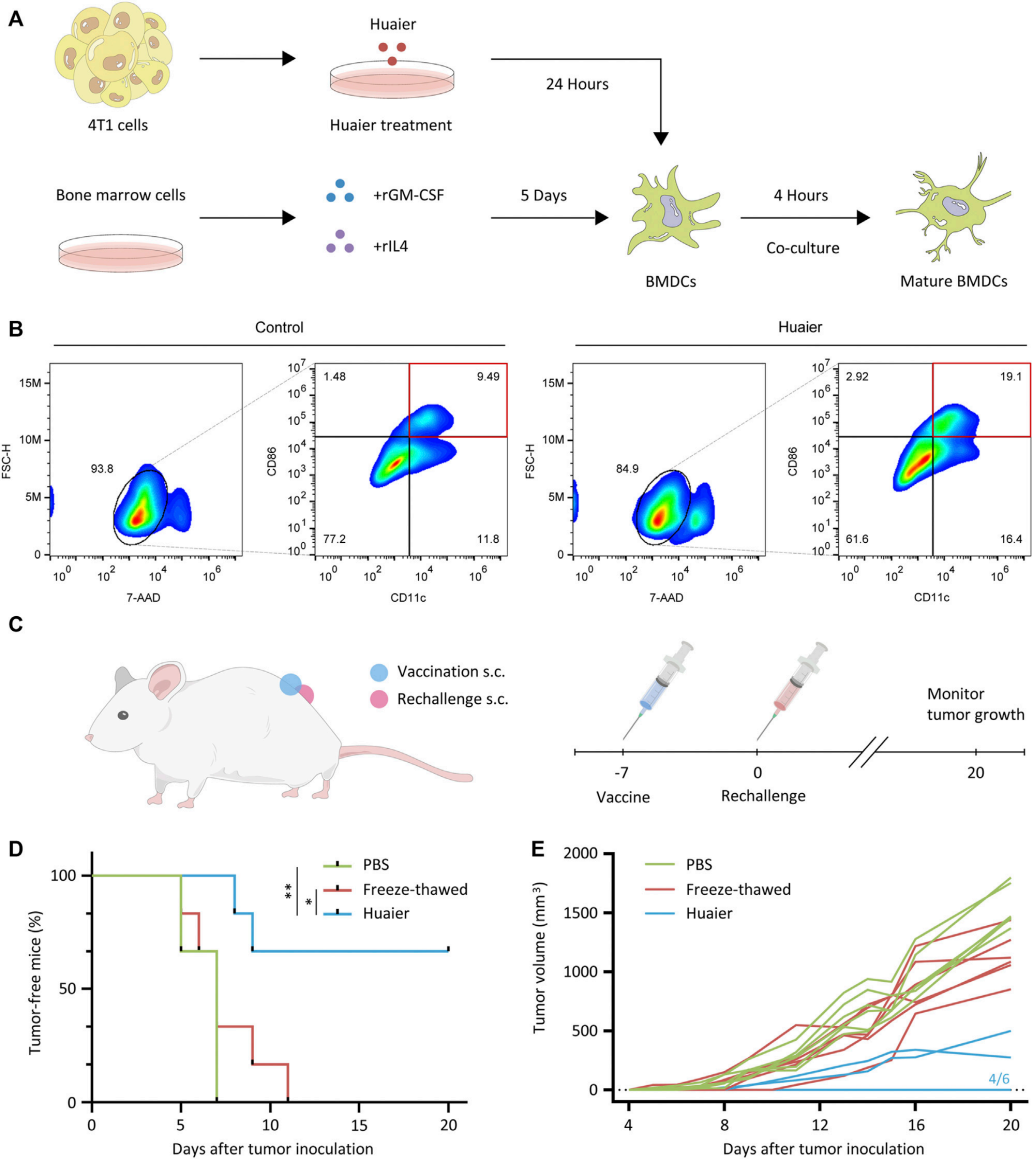
Huaier enhances activation of DC cells. **(A)** The experimental design and cell culture process of bone marrow derived DC cells. **(B)** The populations of activated DCs (CD86^high^ CD11c^+^) were presented via the FlowJo analyses. **(C)** Scheme of the vaccination assay *in vivo*. **(D)** 4T1 cells were pretreated with Huaier, or freeze-thawed, followed by inoculated s.c. into BALB/c mice as a vaccine (*n* = 6 for each group). A week later, mice were rechallenged with live 4T1 cells. **(E)** Individual tumor growth curves of tumor-bearing mice in three groups. s.c., subcutaneously; *p < 0.05; **p < 0.01.

DCs are a family of specialized APCs that are essential to the initiation of antigen-specific tumor immune responses (Ardavín et al., 2004). For the indispensable role of DCs in the acquisition and presentation of DAMPs related to ICD, we then detected the maturation status of DCs co-cultured with Huaier-treated 4T1 cells. The scheme was exhibited in the Figure 2A. After co-culture with Huaier-treated TNBC cells, compared to the control group, the surface expression of activation marker CD86 on CD11c^+^ mouse BMDCs was enormously augmented (Figure 2B). To further validate the *in vitro* results, we evaluated the immunogenicity of Huaier-treated tumor cells in a vaccination setting based on the 4T1 mouse model. As shown in the Figure 2C, immunocompetent BALB/c mice were vaccinated with PBS, freeze-thawed Huaier-treated or untreated 4T1 cells into the left flank. A week later, the mice were rechallenged by the live 4T1 cells in the right flank and were monitored for tumor growth during 20 days. The mice immunized with Huaier-treat 4T1 cells showed strikingly prolonged tumor-free survival compared with the control groups (Figure 2D). Besides, tumor-bearing mice in the Huaier group also showed distinctly lower tumor volumes than the control groups (Figure 2E). These results verified the tumor immunogenicity elicited by Huaier.

### Huaier Treatment Enhances Infiltration of Immune Cells

**FIGURE 3:**
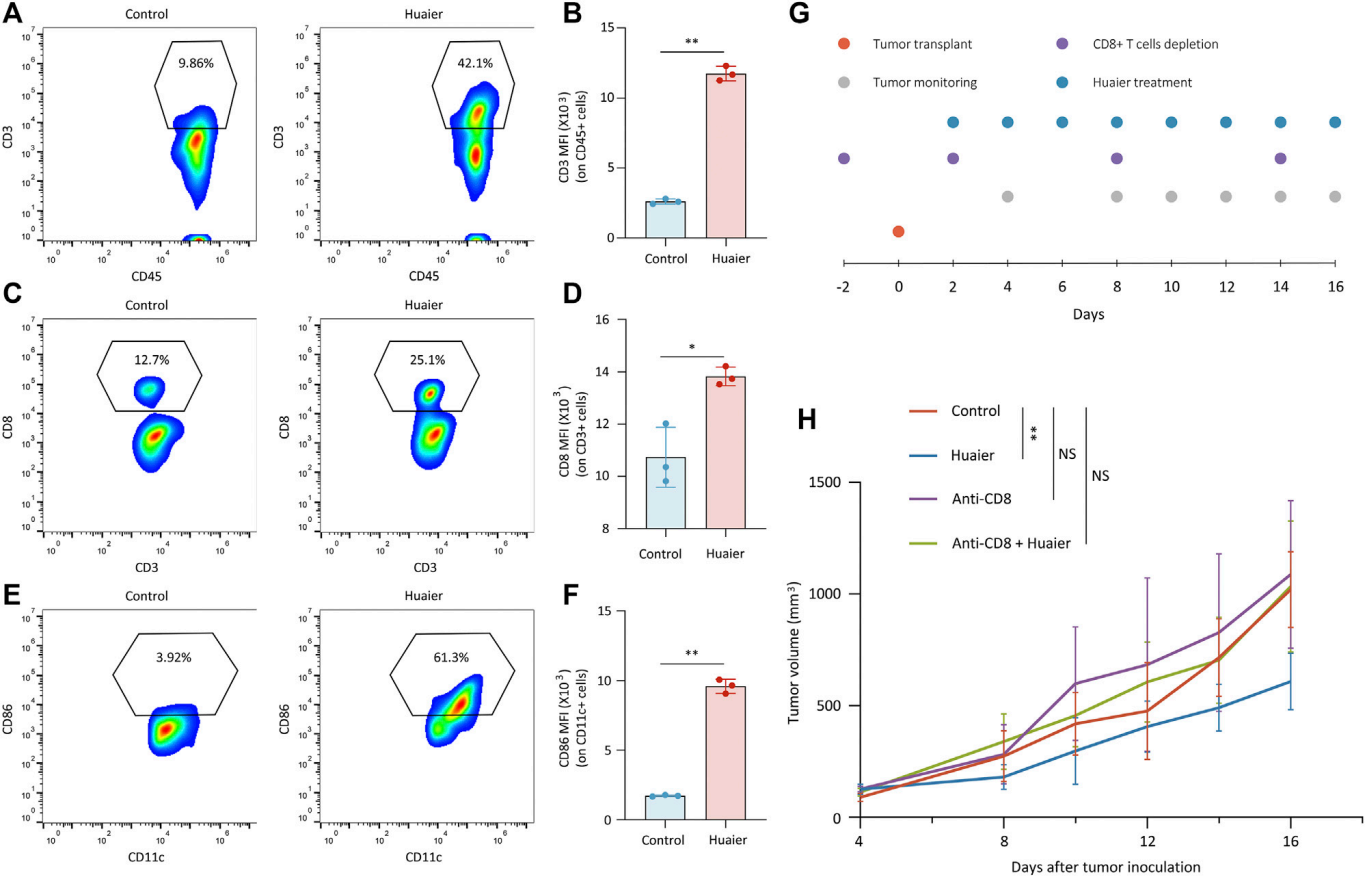
Huaier enhances the infiltration and activation of immune cells *in vivo*. Tumor infiltrating cells were analyzed by flow cytometry. Shown are the population of tumor-infiltrating **(A)** CD3^+^ cells in CD45^+^ cells, **(B)** MFI of CD3 on CD45^+^ cells, **(C)** CD8^+^ cells in CD3^+^ cells, **(D)** MFI of CD8 on CD3^+^ cells, **(E)** CD86^+^ cells in CD11c^+^ cells. **(F)** MFI of CD86 on CD11c^+^ cells. **(G)** Schematic representation of the experimental protocols. **(H)** Average tumor size for treated and untreated tumors in the syngeneic mouse model. Tumor volume was shown as mean ± SD. *n* = 6 per group. MFI, mean fluorescence intensity; *p < 0.05; **p < 0.01.

Previous findings demonstrated that Huaier could induce the immunogenicity of cancer cells, which prompted us to assess the effects of Huaier treatment on the TME via a Balb/c mouse model. Mice bearing 4T1 tumors were orally administrated with Huaier. Tumors were harvested and infiltrating cells were analyzed by flow cytometry (n = 3 per group). The results showed that Huaier treatment radically increased the frequency of tumor infiltrating T cells (Figures 3A,B) and CD8^+^ T cells (Figures 3C,D). Besides, Huaier showed remarkable effects on the activation of DCs (Figures 3E,F). Next, *in vivo* assay was performed to verify the role of CD8^+^ T cells in the treatment of Huaier. The scheme was visualized in the Figure 3G. As shown in the Figure 3H, Huaier considerably impeded the growth of tumors *in vivo*. However, the effect could be attenuated by depletion of CD8^+^ T cells. Thus, the adaptive immunity mediated by CD8^+^ T cells was required in the Huaier induced tumor suppression.

### Huaier Activates Endoplasmic Reticulum Stress Signaling Pathway

**FIGURE 4:**
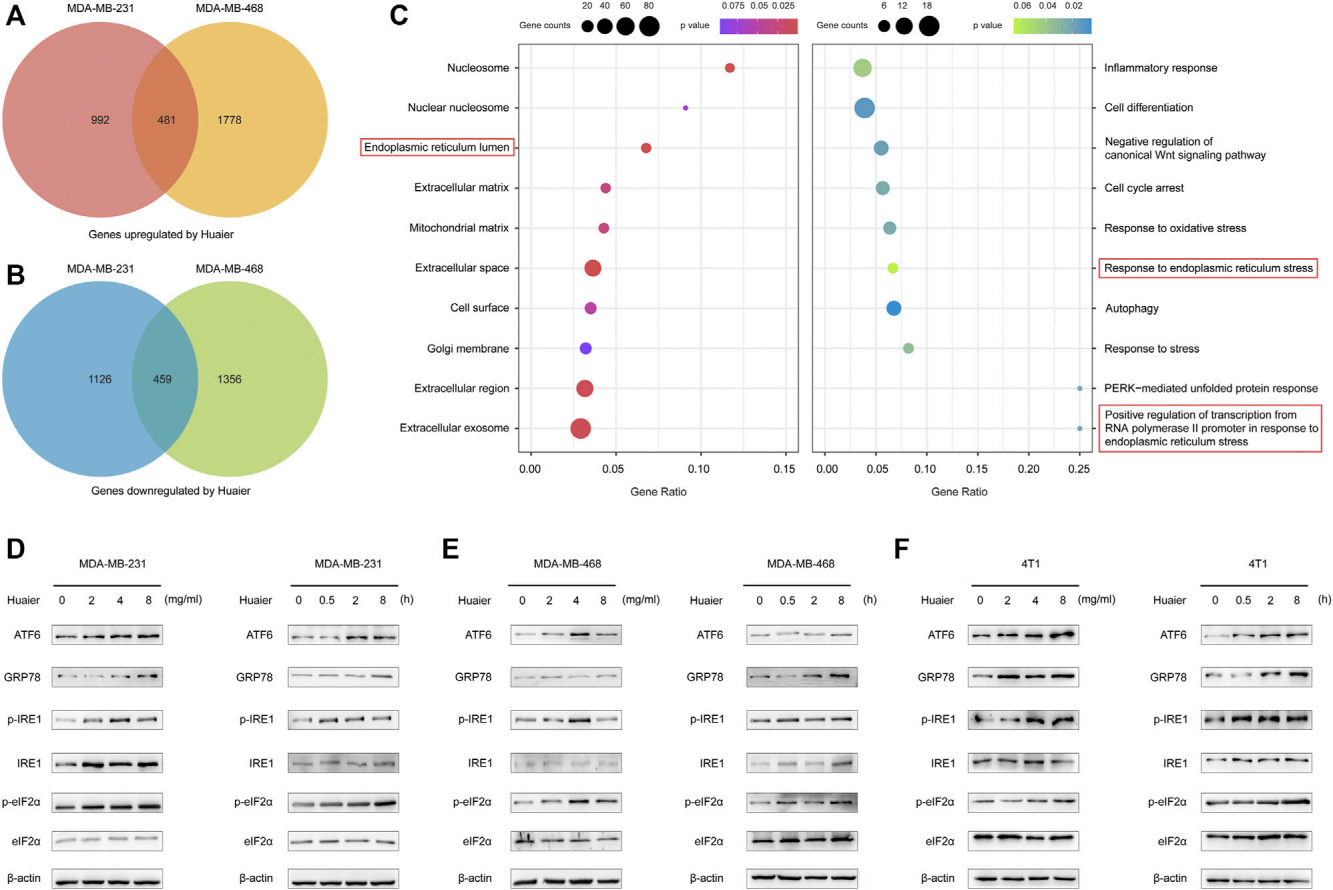
Huaier elicits activation of ER stress signaling pathway in TNBC. **(A,B)** Venn diagram illustrates the up- and down-regulated mRNAs with or without the treatment of Huaier in MDA-MB-231 and MDA-MB-468 cells. **(C)** Functional enrichment analysis of the differentially expressed genes. **(D–F)** Western blotting of ER-stress signaling pathway associated proteins in Huaier treated TNBC cells.

Gene expression profiles was utilized to screen the differentially expressed mRNAs. The criteria was set as fold change > 2. As shown in the Figures 4A,B, 1,473 genes were up-regulated after Huaier treatment, and 1,585 genes were down-regulated in the MDA-MB-231 cells. Expression level of 2259 genes were increased in the MDA-MB-468 cells, while 1815 genes were deceased. The 481 co-upregulated genes and 459 co-downregulated genes were filtered out for the further study.

GO analysis was applied to detect the biological function of the differentially expressed genes. As shown in the Figure 4C, in terms of the cellular component, the up-regulated mRNAs after Huaier treatment were strikingly related with the ER lumen. Furthermore, in the analysis of the biological process, these down-regulated mRNAs were shown to be enriched in the response to endoplasmic reticulum stress and positive regulation of transcription from RNA polymerase II promoter in response to endoplasmic reticulum stress.

Mounting evidence revealed that the effectiveness of various anti-cancer drugs and treatments to induce ICD was relied on the activation of ER stress (Krysko et al., 2012). Western blot was applied to detect the expressions levels of ER stress-associated proteins after Huaier treatment in MDA-MB-231, MDA-MB-468 and 4T1 cell lines. As shown in Figures 4D–F, activating transcription factor 6 (ATF6) and glucose-regulated protein 78 (GRP78; also known as BiP) were sharply up-regulated. Meanwhile, the phosphorylation levels of inositol-requiring enzyme 1 (IRE1) and eukaryotic translation initiation factor 2α (eIF2α) were also augmented upon Huaier treatment.

### Characterization of circCLASP1 in Breast Cancer Cells

**FIGURE 5:**
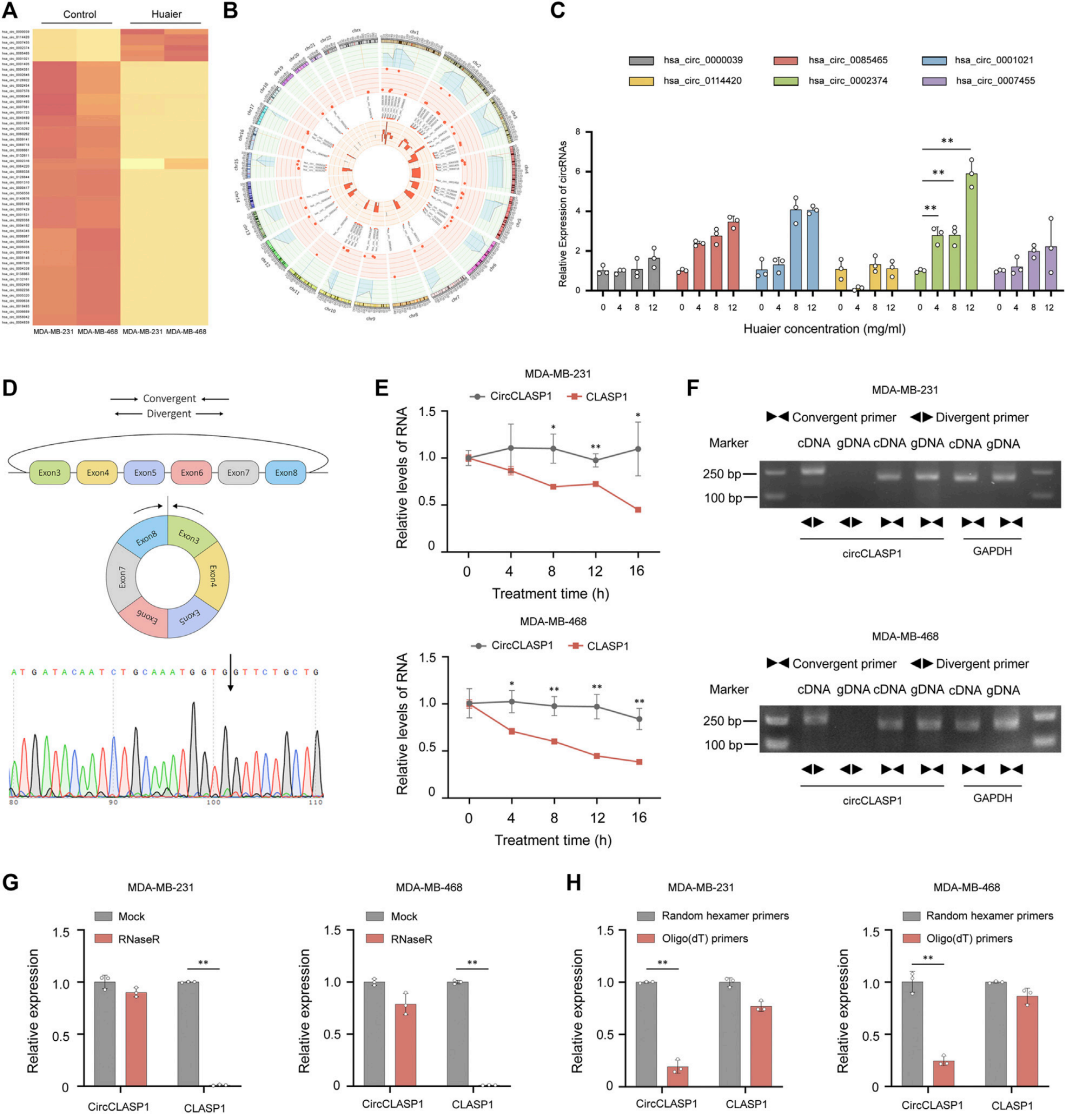
CircCLASP1 is a candidate regulator of Huaier-induced ICD. **(A)** The heatmap showing the up- and down-regulated circRNAs in TNBC cells. **(B)** The differentially expressed circRNAs on human chromosome were visualized in the circos plot. The outermost circle showed the chromosomal distribution of candidate circRNAs. The second circle presented the *p* values. The third circle demonstrated the fold change between two groups. The fourth circle was used to exhibit the circBase IDs of selected circRNAs, and the innermost circle indicated the circRNA expression levels. **(C)** Validation of up-regulated circRNAs in Huaier-treated MDA-MB-231 cells by qRT-PCR. **(D)** CircCLASP1 is derived from exon 3 to 8 of CLASP1 gene. The backsplice junction site of circCLASP1 was identified by Sanger sequencing (black arrow). **(E)** qRT-PCR analysis of circCLASP1 and CLASP1 in Actinomycin D treated MDA-MB-231 and MDA-MB-468 cells. **(F)** CircCLASP1 could be amplified by divergent primers in cDNA but not gDNA. CLASP1 could be amplified by convergent primers in both cDNA and gDNA. GAPDH was used as a linear control. **(G)** The RNA levels of circCLASP1 and liner CLASP1 was detected by qRT-PCR in MDA-MB-231 and MDA-MB-468 cells treated with or without RNase R. **(H)** qRT-PCR analysis of relative expression levels of circCLASP1 and CLASP1 using random hexamer primers and oligo (dT) primers, respectively. *p < 0.05; **p < 0.01.

As an emerging type of noncoding RNAs, circRNAs were extensively detected in mammalian cells. Increasing evidence revealed that circRNAs played a pivotal part during the progression of various malignancies (Chen et al., 2021). To identify the transcripts regulated by Huaier treatment, circRNA expression profile was substantially analyzed and the thresholds were set as fold change >3, *p* < 0.05. 55 differentially expressed circRNAs were filtered out. The expression levels of 6 circRNAs were dramatically higher in the Huaier treated breast cancer cell, while 49 circRNAs were remarkably down-regulated (Figure 5A). Circos plot was used to exhibit the selected circRNAs on the human chromosome (Figure 5B). The outermost circle exhibited the chromosomal distribution of circRNAs. The second circle showed the *p* values of circRNAs. The third circle showed the fold change of indicated circRNAs between two groups. The fourth circle demonstrated the circBase IDs of selected circRNAs. And the innermost circle indicated the gene expression levels of selected circRNAs. Then, we adopted qRT-PCR to validated the 6 up-regulated circRNAs. As shown in the Figure 5C, the expression level of hsa_circ_0002374, could be drastically augmented by Huaier in a dose-dependent manner. Thus, we hypothesized that hsa_circ_0002374 might work as a downstream factor of Huaier extract.

Hsa_circ_0002374 is the circularized product which generated from exon 3 to 8 of CLASP1 gene with a length of 517 nt. Thus, we designated it as circCLASP1. The back-spliced junction of circCLASP1 was amplified using divergent primers and confirmed by Sanger sequencing (Figure 5D). To confirm the covalently closed loop structure of circCLASP1, we next evaluated the stability of circCLASP1 and CLASP1 in MDA-MB-231 and MDA-MB-468 cells treated with Actinomycin D, an inhibitor of transcription. As shown in Figure 5E, the half-life of this circRNA transcript exceeded 16 h, whereas that of the linear form was less stable. PCR analysis for cDNA and gDNA showed that circCLASP1 could be amplified by divergent primers in cDNA but not from gDNA (Figure 5F). Moreover, resistance to digestion by RNase R exonuclease further validated the circular form of circCLASP1. The levels of CLASP1 linear isoform were used to illustrate the efficacy of RNase R treatment (Figure 5G). Additionally, PCR analysis demonstrated that circCLASP1 could be amplified by divergent primers in cDNA reverse-transcribed from random hexamer primers, but not oligo (dT) primers (Figure 5H). These results indicated that circCLASP1 is an abundant and stable circRNA expressed in TNBC cells.

### The Biological Functions of circCLASP1

**FIGURE 6:**
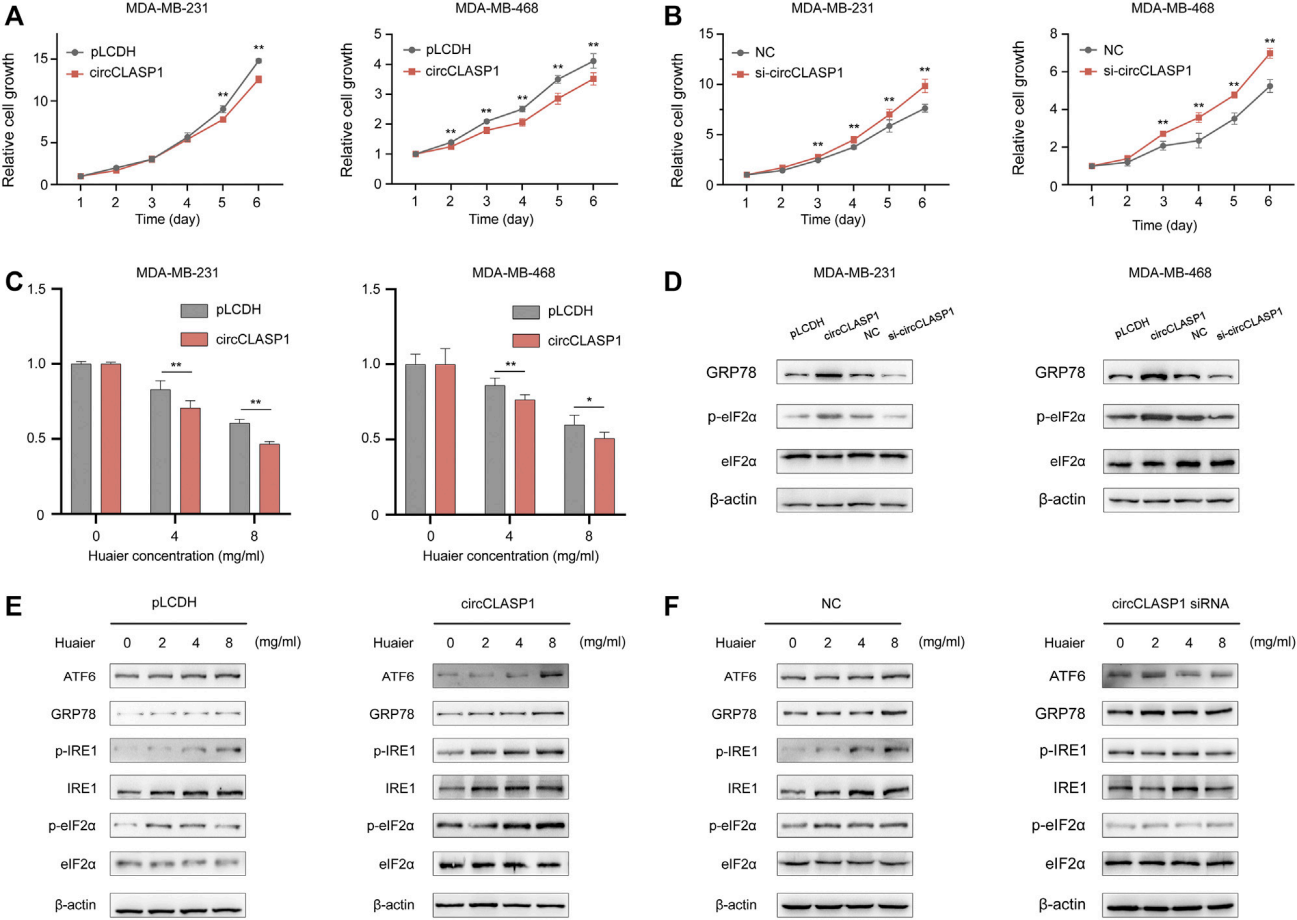
The functional role of circCLASP1 in TNBC. **(A B)** CircCLASP1 drastically attenuated the proliferation of TNBC cells. **(C)** CircCLASP1 enhanced the tumor suppression effects of Huaier in TNBC cells. **(D)** CircCLASP1 elicited ER stress in TNBC cells. **(E,F)** CircCLASP1 promoted Huaier-induced ER stress. *p < 0.05; **p < 0.01.

Gain-of-function and loss-of-function assays were applied to explore the biological functions of circCLASP1 in the MDA-MB-231 and MDA-MB-468 cell lines. The overexpression efficiency of the circCLASP1 were detected by qRT-PCR (Supplementary Figure S1A). To silence circCLASP1, three siRNAs targeting the junction region were applied. As shown in Supplementary Figure S1B, siRNA-1 showed the best knock-down effect. Therefore, siRNA-1 was utilized in the subsequent biological experiments. As shown in Figures 6A,B, up-regulation of circCLASP1 could considerably impede the proliferation of the cancer cells, while knockdown of circCLASP1 showed the opposite effect. Moreover, overexpressing circCLASP1 could dramatically ameliorate the sensitivity of breast cancer cells to Huaier treatment (Figure 6C). Then, to validate the correlation between circCLASP1 and Huaier-induced ER stress, we evaluated the expression levels of the ER stress-associated proteins. Notably, the phosphorylated eIF2α was strikingly promoted after the circCLASP1 transfection (Figure 6D).

To further validate the functional role of circCLASP1 in Huaier induced ICD, we detected ER stress-associated proteins in breast cancer cells based on manipulation of the expression level of circCLASP1. As shown in Figures 6E,F, overexpressing circCLASP1 vastly enhanced the stimulating effects of Huaier, while downregulating circCLASP1 by siRNA could deplete the influence.

### Huaier Enhances the Activation of ER Stress via circCLASP1/PKR/eIF2α Axis

**FIGURE 7:**
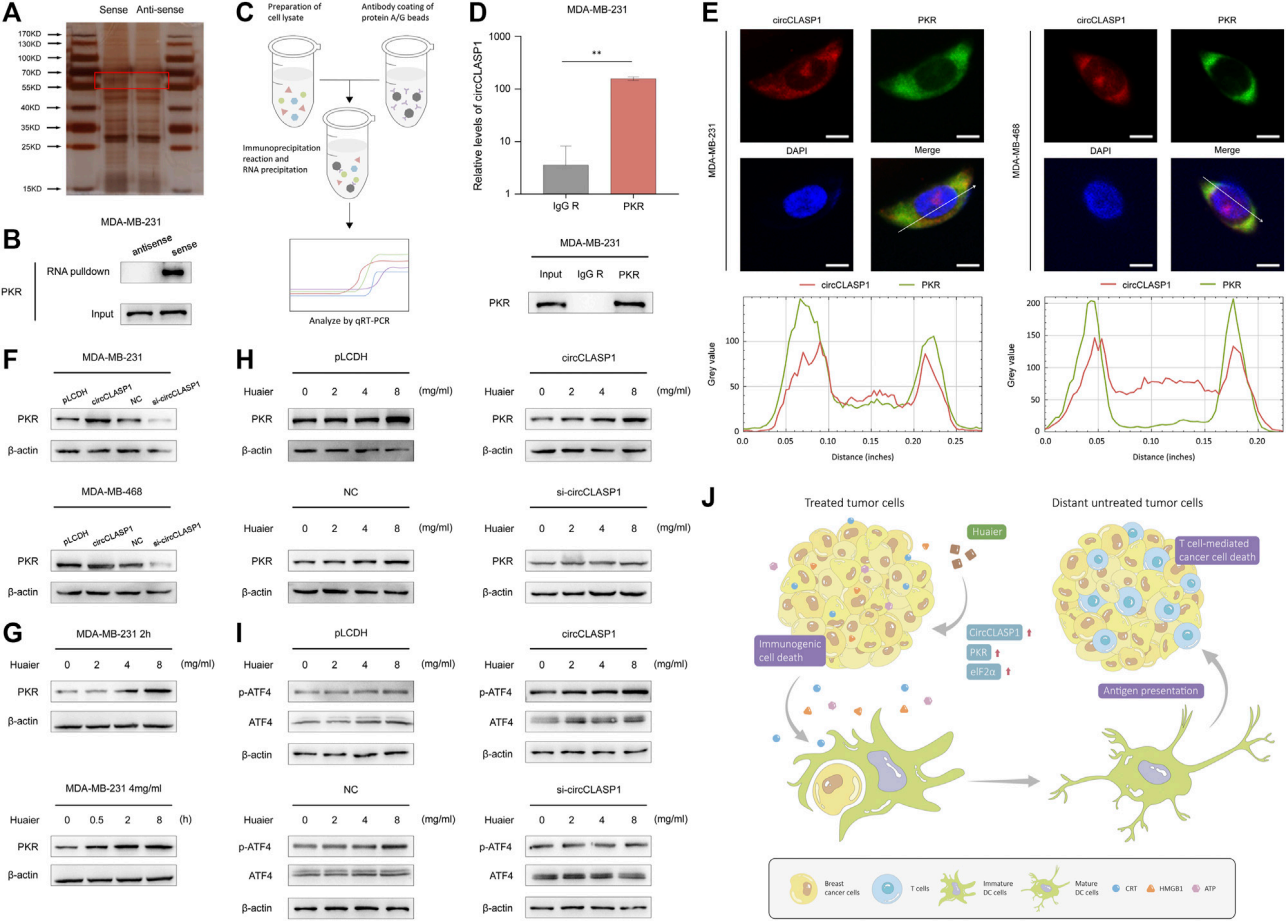
CircCLASP1 enhanced the Huaier-induced ER stress via directly interacting with PKR. **(A)** Silver staining image of proteins pulled down by circCLASP1. **(B)** RNA pull-down and western blotting analyses with sense and anti-sense of circCLASP1 evaluated the physical interaction between circCLASP1 and PKR. **(C)** Schematic representation of the experimental protocols of RIP assay. **(D)** RIP assay was performed using MDA-MB-231 cell lysates and PKR antibodies. Enrichment of circCLASP1 was measured via qRT-PCR analysis. **(E)** FISH and immunofluorescence assays estimated the colocalization of circCLASP1 and PKR in MDA-MB-231 and MDA-MB-468 cells. Scale bars: 10 μm. **(F)** CircCLASP1 enhanced the stabilization of PKR in MDA-MB-231 and MDA-MB-468 cells. **(G)** Huaier increased the protein level of PKR. **(H)** CircCLASP1 enhanced the regulation effects of Huaier on PKR. **(I)** CircCLASP1 enhanced the regulation effects of Huaier on phosphorylation of ATF4. **(J)** Huaier induced immunogenic cell death by circCLASP1/PKR/eIF2α axis in TNBC. *p < 0.05; **p < 0.01.

Subsequently, RNA pulldown assay was applied to find circCLASP1-interacting proteins in MDA-MB-231 cells. As shown in Figure 7A, an obvious band ranging from 55 to 70 kDa was specifically enriched by circCLASP1. PKR was then detected via MS. RNA pulldown assay was utilized to further verify the direct interaction between circCLASP1 and PKR (Figure 7B). RIP assay was used to validate the results (Figures 7C,D). Also, the co-localization of PKR and circCLASP1 was confirmed via FISH and immunofluorescence assay in both MDA-MB-231 and MDA-MB-468 cells (Figure 7E).

According to previous reports, PKR could elicit ER stress via phosphorylation of eIF2a and subsequently activation of ATF4 (Pakos-Zebrucka et al., 2016). Western blot assays were applied to reveal that overexpression of circCLASP1 efficiently enhanced the stabilization of PKR. After knockdown circCLASP1 with siRNA in TNBC cells, the expression of PKR was downregulated (Figure 7F). As shown in Figure 7G, after Huaier treatment, expression level of PKR was noticeably enhanced. Consistently, overexpressing circCLASP1 in TNBC cells could substantially facilitate the function of Huaier, while transfection of circCLASP1 siRNA showed the opposite effect (Figure 7H). PKR/eIF2α/ATF4 axis was previously reported as an important signaling pathway in the activation of ER stress (Pakos-Zebrucka et al., 2016). Thus, we further assessed the expression and phosphorylation level of ATF4 after Huaier-treatment. As exhibited in the Figure 7I, phosphorylation of ATF4 could be enhanced by Huaier in a dose-dependent manner, which could be augmented via circCLASP1 overexpression. Consistently, transfection of circCLASP1 siRNA in TNBC cells attenuated this effect. Taken together, circCLASP1/PKR/eIF2α axis played an indispensable role in Huaier induced ER stress and ICD (Figure 7J).

## Discussion

Breast cancer was previously classified into immunologically silent neoplasms (Emens et al., 2021). Due to the higher levels of TILs in the TME, TNBC is recently recognized as an immunogenic subtype of breast cancer (Kwa and Adams, 2018). Thus, cancer immunotherapy has become the focus of attention in the treatment of TNBC. Nevertheless, the objective response rate (ORR) in TNBC is unsatisfied so far (Voorwerk et al., 2019). ICD was considered to be involved in the augmented production of immune-activating stimuli and enhanced presentation of TAAs (Wang et al., 2019). Therefore, utilizing certain agents to elicit ICD could be an encouraging strategy to alter the immune-deserted neoplasm into immune-cultivated ones. Thus, in the present study, the ICD-inducing effects of Huaier were systematically studied.

The immunogenic features of ICD are largely mediated by DAMPs, such as emission of ATP, secretion of HMGB1 as well as cell surface exposure of CRT (Krysko et al., 2012). Intracellular ATP has long been known as a vital energy-carrying molecule produced through mitochondrial oxidative phosphorylation (OXPHOS) and glycolysis (Bennett et al., 2020). Meanwhile, the released ATP exerts a chemotactic effect for DCs via binding to the purinergic receptor P2Y2 (P2RY2), thus facilitating the presentation of TAAs (Chekeni et al., 2010). HMGB1 was previously considered as a highly conserved non-histone chromatin-binding protein which worked as a DNA chaperone. In the recent years, the presence of HMGB1 in the extracellular space has been reported (Sekiguchi and Kawabata, 2020). With redox modifications on cysteine residues, HMGB1 could exert a chemotactic activity to initiate immune response through synthesizing a heterocomplex with CXCL12 (Yang et al., 2013). CRT is a calcium-binding protein which mainly exists in the ER. When displayed on the cell surface of dying tumor cells, CRT could also act as a specific protein marker recognized by DCs which tags them for elimination by the immune system (Obeid et al., 2007). In our research, Huaier treatment could lead to enormous up-regulation of CRT exposure in the cell membrane of TNBC cells. Also, release of ATP and HMGB1 were enormously enhanced in the cell culture supernatants.

As the main sentinel APCs in the immune system, DCs were recognized as a pivotal connect between innate and adaptive immune responses (Lamberti et al., 2020). After phagocytosis of TAAs, the immature DCs would undergo a maturation process. Meanwhile, the antigens were processed into small bioactive peptides to synthesize MHC-peptides complexes (Duong et al., 2022). Mature DCs then shift from peripheral tissue to secondary lymphoid organs, where they could present the TAAs to helper T cells or effector T cells, thus triggering tumor-specific immune responses (Leone et al., 2018). Due to the effective role of DCs, cell-based vaccine emerged as a promising option in tumor treatment. According to Huang et al.(Huang et al., 2022), a *in situ* DC-primed vaccine drastically facilitated CD8^+^ T cell responses, which resulted in obvious tumor suppression in TNBC mouse xenograft model and patient-derived tumor organoids. In our study, we co-cultured BMDCs with Huaier-treated or -untreated 4T1 cells, the results revealed that TNBC cells could provoke the activation of DCs in the presents of Huaier. Consistently, *in vivo* assay verified the result that Huaier could strikingly prolong the tumor-free survival and suppress TNBC growth when used in a vaccine setting. Furthermore, oral administration of Huaier efficiently increased the percentage of mature DCs in the tumor bed.

High levels of TILs were considerably correlated with lower recurrence or mortality, and predicted superior overall survival in TNBC patients (Ibrahim et al., 2014). As a subset of TILs, CD8^+^ T cells are recognized as major drivers of antitumor immunity. Abundance of CD8^+^ T cell infiltration was reported to serve as a potentially predicting biomarker for response to immune checkpoint therapy in TNBC (Oshi et al., 2020). Besides, Telli et al.(Telli et al., 2021) showed that Tavo treatment could sensitize TNBC patients to anti-PD-1 therapy via promoting the infiltrating of CD8^+^ T Cells. In this research, *in vivo* assays showed that gavage treatment of Huaier could vastly facilitate the infiltration of CD8^+^ T cells in the TME. Moreover, specifically depleting CD8^+^ T cells could rescue the tumor suppress effects of Huaier in the TNBC-bearing mouse model.

The ER was a pivotal structure in the cell, which governing lipid biosynthesis, calcium storage as well as protein translation and modification. However, numerous cell-intrinsic events and external factors could impede the process of protein synthesis in the ER (Oakes and Papa, 2015). Accumulation of unfolded or misfolded proteins in the organelle consequently leads to ER stress. Moderate ER stress could maintain ER homeostasis to enhance cellular survival under stress. Nevertheless, excessive or persistent ER stress could trigger programed cell death (Chen and Cubillos-Ruiz, 2021). Recent years, YD277, a novel small molecule, was proved to be able to elicit G1 cell cycle arrest and lead to apoptosis in multiple breast cancer cell lines by activating ER stress pathway (Chen et al., 2017). It was also reported that inhibition of SGK3, a downstream kinase of ERα in breast cancer, could drastically suppress aromatase inhibitors (AI)-resistant tumor cell survival via inducing intense ER stress (Wang et al., 2017). Moreover, accumulating evidence revealed a key role for ER stress response in the process of ICD. According to Li et at. (Li et al., 2021), oleandrin, a cardiac glycoside, could elicit an ER stress-related ICD through PERK/eIF2α/ATF4/CHOP signaling pathway. Statin-induced suppression of KRAS prenylation could cause dramatic ER stress via KRAS mutation, thus driving ICD of KRAS^mut^ cancer cells (Nam et al., 2021). Here, we evaluated the gene expression profiles of Huaier-treated TNBC cells via GO analysis. The results showed that Huaier could be closely associated with ER stress. Biological experiments were then applied to validate the hypothesis. After Huaier treatment, the expression of ER-stress related proteins was remarkably promoted. Notably, Huaier substantially facilitated the phosphorylation of eIF2α, indicating that Huaier-induced ICD mainly depend on the ER-stress signaling pathway.

Noncoding RNAs (ncRNAs) (primarily microRNAs, long noncoding RNA, and circular RNA) are a majority of the transcripts which do not possess obvious protein-coding ability (Sun and Chen, 2020). During the past decade, concept on ncRNAs had concerted from “junk” transcriptional sequences to key regulators mediating various biological processes (Anastasiadou et al., 2018). Recently, studies revealed an emerging role of ncRNAs on manipulating the exposure of ICD-associated DAMPs (Lamberti et al., 2021). MiR-27a was reported to attenuate the sensitivity to drug-stimulated ICD via the regulatory axis with CRT (Colangelo et al., 2016). Combination of miR-200c and DOX in the nanoparticles could efficiently suppress the expression of PD-L1 and elicit ICD in tumor cells (Phung et al., 2019). CircRNAs are a newly identified class of noncoding RNAs with closed continuous loop structure. Recent years, mounting evidence indicated a functional role of circRNAs in the modulation of immune system (Zlotorynski, 2019). Circular RNA circ_0020710 could regulate the miR-370-3p/CXCL12 axis and thus lead to immune evasion (Wei et al., 2020). Besides, circMET showed an enormous immunosuppression effect through enhancing the Snail/DPP4/CXCL10 signaling pathway (Huang et al., 2020). However, the function of circRNAs in the regulation of ICD has not been discovered yet. In this study, through analyzing the differentially expressed circRNAs, circCLASP1 was proved to be the downstream factor of Huaier. Gain- and loss-of-function assays showed that circCLASP1 could remarkably up-regulate the ER signaling pathway and improve the sensitivity to Huaier treatment in TNBC cells through ICD. PKR is a double-stranded RNA-activated protein kinase. Previously studies have demonstrated its multifaceted roles in cancer, including proliferation, drug resistance and innate immune response (Lee et al., 2020). Novel discovered evidence indicated that certain circRNAs could directly interact with proteins. circSHKBP1 markedly stabilized HSP90 via blocking STUB1-mediated ubiquitylation (Xie et al., 2020). In the present research, circCLASP1 could interact with PKR and effectively protected it from degradation, which further phosphorylated eIF2α at Ser51 and activated ER stress.

## Conclusion

In the present study, we report that Huaier, an extract of *Trametes robiniophila Murr*, inhibits TNBC progression through eliciting ICD and resulting in facilitated immunogenicity of cancer cells. Furthermore, the augmented tumor-suppressing efficacy was largely dependent on the CD8^+^ TILs. The therapeutic effects of Huaier are closely associated with ER stress *via* circCLASP1/PKR/eIF2α axis. Our findings revealed a promising role of traditional Chinese medicine in the immune stimulation and provided supporting evidence for the application of Huaier as a potential ICD-inducer in the treatment of TNBC patients.

## Data Availability

The datasets presented in this study can be found in online repositories. The names of the repository/repositories and accession number(s) can be found in the article/Supplementary Material.
