# Supplementary material for: Huaier Induces Immunogenic Cell Death Via CircCLASP1/PKR/eIF2α Signaling Pathway in Triple Negative Breast Cancer
Source: Front Cell Dev Biol. 2022 Jun 16;10:913824. doi: 10.3389/fcell.2022.913824 (PMC9243662; doi:10.3389/fcell.2022.913824)
Supplement: Supplementary file 1 [file DataSheet1.DOCX]

Supplementary Material

# Supplementary Figures





**Supplementary Figure 1.** The overexpressing and knock-down efficiency of the circCLASP1. (A) qRT-PCR detection of the expression levels of circCLASP1 after transfection with circCLASP1 vectors in MDA-MB-231 and MDA-MB-468 cells. (B) Expression levels of circCLASP1 treated with siRNAs in MDA-MB-231 and MDA-MB-468 cells. **p* < 0.05; ***p* < 0.01

# Supplementary Tables

## Supplementary Table 1. Primer sets used for qRT-PCR

| **Gene name** | **Primers** | **Sequence (5’-3’)** |
| --- | --- | --- |
| circCLASP1 | Forward | TACAGACATGTAGGAGAACGTGTGAG |
|  | Reverse | CTTTAGCATCTCCTAGTCTGTCTATTAGACT |
| CLASP1 | Forward | GTGGTGAGAGCGGCTGA |
|  | Reverse | CAGCAAGCCTGGGATGATGT |
| hsa_circ_0000039 | Forward | ACTGCGACACATTCGCCTAG |
|  | Reverse | CTAGGGCTCCTGGTTGCC |
| hsa_circ_0085465 | Forward | ACCACCTGAGCCAAGATCAC |
|  | Reverse | TGCTACTGGGAGTACCTCCAAA |
| hsa_circ_0001021 | Forward | GCAGGCTACTGAATCTCATCATCG |
|  | Reverse | GTTTGAAGGTACAAACTCTTCCTTGGG |
| hsa_circ_0114420 | Forward | GGCCGAAGCAGCTTCATAGA |
|  | Reverse | GGCTGATCCACAGGCTGT |
| hsa_circ_0007455 | Forward | GATGCTGATGCTGATGAAAACTTTT |
|  | Reverse | TGAAAGAATATTTGCATTTCTTTGACTT |
| β-Actin | Forward | CATGTACGTTGCTATCCAGGC |
|  | Reverse | CTCCTTAATGTCACGCACGAT |
| GAPDH | Forward | GGAGCGAGATCCCTCCAAAAT |
|  | Reverse | GGCTGTTGTCATACTTCTCATGG |

## Supplementary Table 2. Sequence of the siRNA used in this study

| Gene name | Sequence (5’-3’) |
| --- | --- |
| circCLASP1 siRNA-1 | CAATCTGCAAATGGTGGTT |
| circCLASP1 siRNA-2 | TGCAAATGGTGGTTCTGCT |
| circCLASP1 siRNA-3 | TCTGCAAATGGTGGTTCTG |

## Supplementary Table 3. Sequence of the FISH probe used in this study

| Gene name | Probe (5’-3’) |
| --- | --- |
| circCLASP1 (FISH) | Cy3-AACCACCATTTGCAGATTGTATCAT |
